# Peripheral artery disease and local drug delivery: a review of disease pathology and drug delivery systems for therapy below the knee

**DOI:** 10.3389/ebm.2025.10754

**Published:** 2025-12-08

**Authors:** Nicole M. Akers, Tammy R. Dugas

**Affiliations:** Department of Comparative Biomedical Sciences, Louisiana State University School of Veterinary Medicine, Baton Rouge, LA, United States

**Keywords:** peripheral artery disease, drug coated balloon, paclitaxel, sirolimus, thrombosis, combination device, biocompatibility

## Abstract

Peripheral artery disease (PAD) is a disease of both atherosclerotic and thromboembolic pathology, affecting more than 230 million people globally. PAD patients are at an increased risk of thrombotic events and often require lifelong antithrombotic therapy. Thromboembolism can lead to complete occlusion of affected arteries and put patients at risk for critical limb threatening ischemia (CTLI). PAD blockages are cleared using drug-eluting stents (DES) and drug-coated balloons (DCB). However, PAD treatment below the knee (BTK) presents unique challenges. While DCB are frequently used to treat BTK disease, no DCB has gained FDA approval for this indication. However, innovation in the field has produced drug delivery systems and formulations that may yet enhance the effectiveness of these therapies. In this review, we will provide a brief overview of the pathological mechanisms associated with PAD and review the materials and drugs frequently used in DCBs with an emphasis on excipients and drug carriers. Finally, we will highlight emerging devices undergoing clinical trials to treat BTK disease and how they differ from their predecessors.

## Impact statement

We provide timely updates to the progress being made in combination device development for peripheral artery disease (PAD) therapy. This review article summarizes both basic pathophysiologic information for PAD as well as device development considerations for combination devices. Lesions below the knee have proven challenging to treat. Drug coated balloons are frequently used as a part of PAD lesion treatment below the knee, yet none are approved for use below the knee in the US. Therefore, we discuss the latest updates in the development of several promising combination and lesion preparation devices for treatment of PAD disease below the knee, a historically recalcitrant area to treat. This information will be useful to both scientists and clinicians who are either developing their own combination devices or looking for cutting edge information on how new devices are different from their predecessors.

## Introduction

Peripheral artery disease (PAD) encompasses atherosclerotic and thrombotic pathology outside of the coronary and cerebral vascular systems. The most common presentation of PAD occurs within the lower limbs, with an estimated global prevalence of more than 230 million cases [1]. PAD is associated with significant morbidity, disability, and mortality in affected individuals. These subjects can experience limb weakness and claudication due to decreased tissue perfusion from narrowed, damaged vessels, up to complete occlusion of blood flow, leading to critical limb-threatening ischemia (CLTI) and potential limb amputation. The risk for myocardial infarction (MI) or stroke in PAD patients is on par with patients suffering from coronary artery disease [2]. PAD treatment includes a combination of lifestyle modifications, medical therapy, and when needed, endovascular interventions including surgical approaches and medical device interventions [3, 4]. There are a wide variety of devices available to treat PAD lesions, including the use of bare-metal stents (BMS) and drug-eluting stents (DES), either balloon-expanded or self-expanding, newer woven and covered nitinol stents, dissolvable scaffolds, percutaneous transluminal angioplasty (PTA) with either drug-coated (DCBs) or plain balloons (POBA), intravascular lithotripsy and atherectomy to treat calcified lesions [5]. Treatment approaches are highly dependent on the location, length and number of lesions present, as well as the pattern of disease in the individual and their comorbidities. While both stenting and balloon angioplasty have been successful above the knee, lesions below the knee (BTK) have restenosis rates that approach 70 percent [6]. Additionally, BTK lesions are heavily calcified, cover extensive lengths of the artery, and importantly, possess significant thromboembolic pathology that can lead to adverse outcomes including CLTI and subsequent amputation [7]. Below the knee, DCBs are commonly used to treat lesioned arteries; yet none are FDA approved for use below the knee due to a lack of evidence of long term benefits over POBA. Recent developments in DCB include novel drug formulations and carriers, which may yet improve clinical outcomes in the long term for BTK disease. These carriers include liposomal formulations, polymeric microspheres, and aqueous delivery systems, among others. Before delving into these novel technologies, we will first discuss PAD pathophysiology, drugs commonly used in DCB, the difficulty associated with BTK disease treatment, and how coating formulations can enhance or derail effective DCB treatment.

## Pathologic mechanisms of PAD

### Endothelial regulation of thrombosis

Healthy endothelial cells express both prostacyclin (PGI_2_) and endothelial nitric oxide synthase (eNOS). eNOS provides a source of nitric oxide (NO), which along with PGI_2_, synergistically inhibits platelet adhesion and aggregation via binding to receptors expressed on the platelet surface, reducing their activity [8]. NO is additionally a vasodilator that permeates the endothelium, promoting relaxation of the vascular smooth muscle. Vascular endothelial cells are key regulators of coagulation and thrombosis. TV-VIIa (activated factor VII complex) and prothrombinase are key initiators of early clot development; vascular endothelial cells express TFPI-ɑ and TFPI-*β* (tissue factor pathway inhibitor alpha and beta, respectively) that inhibit the TF-VIIa (activated factor VII) complex and prothrombinase. Therefore, TFPI-ɑ and TFPI-*β* inhibit clot formation at an early stage [9]. Fibrinolysis is regulated via plasminogen activator inhibitor (PAI-1), endothelial urokinase plasminogen activator (u-PA) and tissue plasminogen activator (t-PA). Thus, under normal circumstances the luminal endothelial surface is antithrombogenic, expressing multiple inhibitors of coagulation, platelet aggregation and adhesion, as well as other factors that promote fibrinolysis. On the other hand, decreased eNOS activity in damaged endothelia promotes platelet aggregation, reactivity, and thrombosis [8].

### Invasion of lipids and inflammatory cells

Inflammatory cytokines, reactive oxygen species, and high levels of circulating LDLs behave as endothelial stressors [10, 11]. Under chronic exposure to stressors, endothelia can become dysfunctional. Dysregulated eNOS activity due to endothelial dysfunction reduces NO output. NO is critical for maintaining endothelial barrier function, and NO inhibits NF-κB, a key transcription factor that promotes the expression of ICAM, VCAM-1, E-selectin, and other leukocyte adhesion receptors on the endothelium [12]. As a result of compromised endothelial barrier function, the endothelium becomes permeable to the transmigration of inflammatory cells and lipids via decreased NO production [13]. Monocytes thus transmigrate through the endothelium and differentiate into macrophages, phagocytizing LDLs. Phagocytosis of LDL transforms macrophages into foam cells. These foam cells become apoptotic and are cleared by M2 macrophages. When these macrophages die, they release TF, lipids, and other inflammatory molecules including matrix metalloproteinases (MMP), further promoting a prothrombotic and inflammatory state [14].

### Atheroma development

Transformation of macrophages into foam cells that deposit lipids into the subendothelial matrix marks the initiation of atheroma development. Like coronary artery disease (CAD), atherosclerotic plaques can be found in PAD lesions. Atherogensis can be defined by several stages, beginning with a fatty streak within the artery wall, progressing to a fibrous plaque, to an unstable plaque at risk of rupture, and ultimately, a ruptured plaque. Atherosclerotic plaques contain a mixture of lipids, minerals, inflammatory cells, platelets and cellular degradation products [15]. Additionally, ruptured atherosclerotic plaques release pro-thrombotic factors such as tissue factor (TF) from the necrotic core, which promotes thrombosis [16].

### Medial calcification

Calcification associated with CAD and calcified lesions associated with PAD differ. Calcification can be classified as intimal or medial depending on where it is located within the vessel tunics, each with different proposed mechanisms for their pathogenesis. While CAD calcification is primarily intimal and associated with fibrous plaques, PAD calcification can be found in both the intima and media [17]. Importantly, medial calcification is often observed with CTLI [7]. Medial calcification was once considered a benign, aging-associated change in the tunica media; however, it may contribute to thrombotic risk. While atherosclerotic plaque rupture is considered a central mechanism for thromboembolism in the presence of plaques, highly calcified BTK lesions with minimal atherosclerotic involvement also display thromboembolic pathology [7]. This suggests the possibility that there are alternative pro-thrombotic mechanisms outside of atherosclerotic plaque rupture. Chang and coworkers proposed lower leg arterial calcification as a potential risk factor for acute thrombosis independent of atherosclerotic pathology [18]. Accordingly, luminal thrombi in BTK lesions are not routinely associated with atherosclerotic plaques compared to CAD lesions but are commonly associated with heavily calcified arteries [19]. Narula et al., examined 299 arteries collected from 95 patients with CTLI. BTK arteries presented more often with diffuse, chronic, occlusive thromboembolic pathology and significant calcification with minimal atherosclerotic disease when compared to FP lesions, although both BTK and FP segments displayed medial calcification [7].

Additionally, different patterns of calcification present in FP and BTK lesions may complicate patient outcomes. FP segments often have thick, patchy calcifications associated with the tunica intima, whereas BTK lesions frequently present with continuous, annular calcifications which form a circumferential ring of arterial calficiation [20]. Annular calcification patterns have been tied to poor long term survival in patients with CTLI [21]. Lastly, intensely calcified arteries pose a significant physical barrier against the transfer of drugs through the arterial wall.

### Mechanisms of vascular remodeling following balloon injury in PAD patients

**FIGURE 1:**
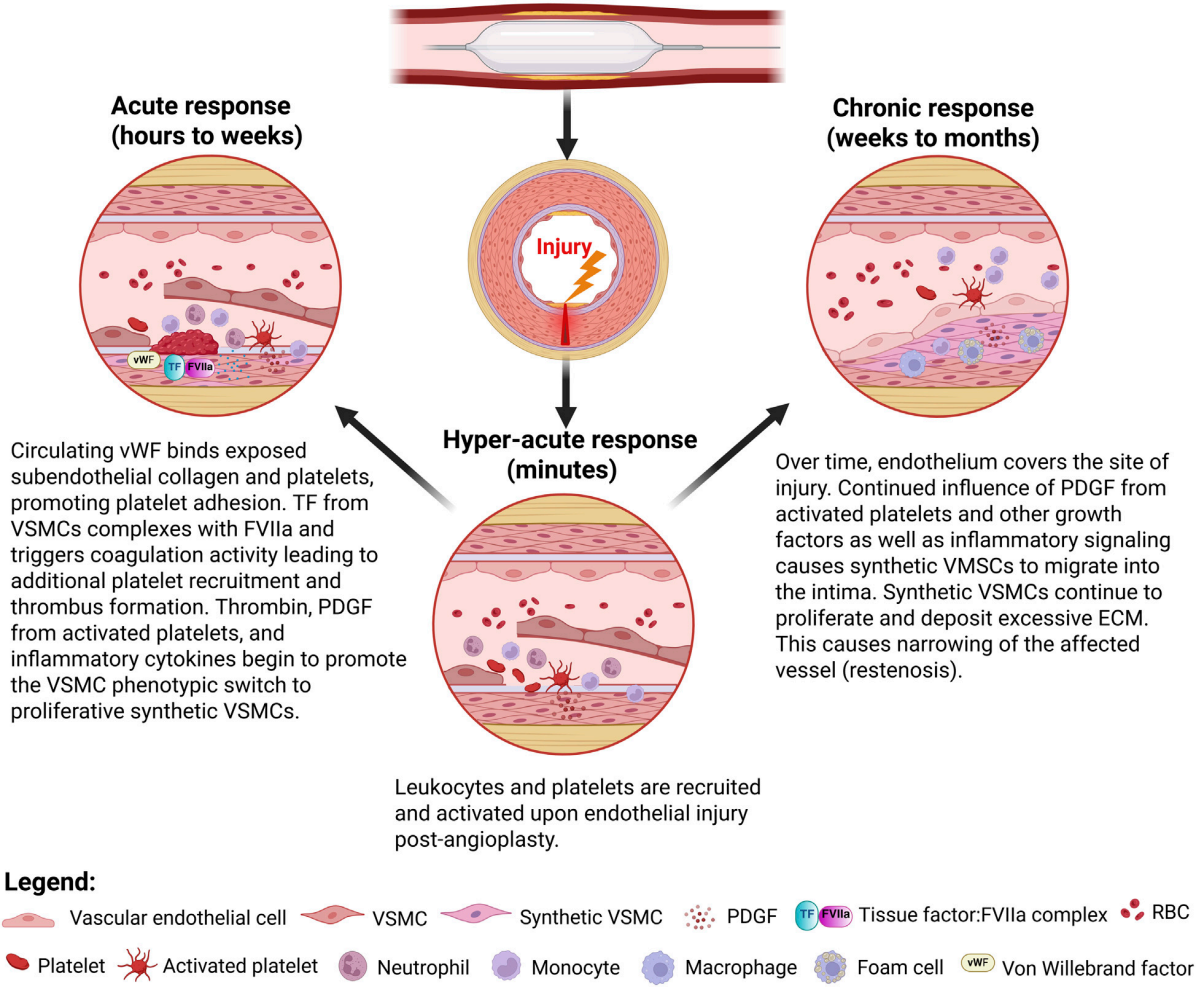
*Vascular responses to balloon angioplasty leading to intimal hyperplasia*. Created in BioRender. Dugas, T. (2025) https://BioRender.com/y22t579.

Balloon angioplasty has become the preferred approach to BTK lesions. However, endothelial damage including complete denudation of the endothelial layer at the site of angioplasty can occur [22]. This damage contributes to IH development, which starts with the recruitment of platelets and inflammatory cells to injured sites and results in the synthetic vascular smooth muscle cells (sVMSC) phenotypic switch that promotes remodeling of the arterial wall. Circulating platelets adhere to exposed subendothelial collagen by binding to vWF and collagen via platelet surface glycoproteins GPVI and GPIb-IX-V [23]. Bound platelets become activated and recruit additional platelets through the release of aggregatory mediators including ADP, thromboxane A_2_, and thrombin [24]. Thrombin generated through TF-mediated extrinsic coagulation activity further bolsters platelet aggregation [25]. Importantly, these bound platelets secrete platelet-derived growth factor (PDGF), a potent activator of VSMC migration and proliferation [26, 27]. Additionally, macrophages have been long established as promoters of the sVSMC phenotype. M1 macrophages, which are often found in association with atherosclerotic lesions and in damaged tissues following balloon angioplasty, secrete numerous cytokines including TNF-α, IL-1B and IL-6 [28]. These cytokines have all been implicated in modulating the VSMC phenotype, enhancing proliferation and migration [29, 30]. Under the influence of growth factors PDGF as well as inflammatory cytokines, VSMC behave as sVSMC. These sVSMC migrate from the tunica media to the tunica intima, where they proliferate and secrete extracellular matrix (ECM) components leading to ECM deposition and expansion [31]. Ultimately, the root of the remodeling process is endothelial injury triggering this cascade of events Figure 1.

## Challenges in treating PAD below the knee

Drug coated devices targeting vascular smooth muscle cell proliferation have improved vascular patency over bare metal stents and uncoated balloons in femoropopliteal lesions [32, 33]. However, long term benefits from DCBs in BTK segments have yet to be established. Below the knee, the infrapopliteal vessels including the anterior and posterior tibial, fibular, and pedal arteries are smaller, thinner, and are exposed to numerous mechanical forces. Stents run an elevated risk of fracture BTK and are typically used as a bailout option. Balloon angioplasty is considered the primary therapy to treat BTK PAD. However, there are multiple success-limiting factors for BTK lesions which include long lesion length, significant calcification, vascular elastic recoil, flow-limiting dissection, and restenosis [34]. While many DCB have gained FDA approval, none are approved for BTK arteries Table 1.

**TABLE 1:** Endovascular combination devices approved by the FDA in the past 10 years for the treatment of PAD.

Device	Manufacturer	FDA approval status	Lesion indication	Drug (μg/mm^2)	Coating
Esprit™ BTK everolimus eluting resorbable scaffold system	Abbott vascular (IDEF technologies inc.)	Approved 4/26/24	BTK	Everolimus 1 μg/mm^2^	Poly (D,L-lactide)
SurVeil drug-coated balloon	Surmodics, inc.	Approved 6/16/23	FP	Paclitaxel 2 μg/mm^2^	Polyethyleneimine polymer
Chocolate touch paclitaxel drug-coated PTA balloon catheter	TriReme medical, LLC, (now genesis medtech)	Approved 11/04/22	FP	Paclitaxel 2.95 μg/mm^2^	Propyl gallate
Ranger™ paclitaxel-coated PTA balloon catheter	Boston Scientific corporation	Approved 10/30/20	FP	Paclitaxel 2 μg/mm^2^	Acetyl tributyl citrate
Eluvia drug-eluting vascular stent system	Boston Scientific corporation	Approved 9/18/18	FP	Paclitaxel 0.167 μg/mm^2^	PBMA (poly (n-butylmethacrylate)) and PVDF-HFP (vinylidene fluoride and hexafluoropropylene copolymer)
Stellarex 0.035 OTW drug-coated angioplasty balloon	The spectranetics corp.	Approved 7/26/17	FP	Paclitaxel 2 μg/mm^2^	PEG-8000
IN.PACT admiral paclitaxel-coated PTA balloon catheter and IN.PACT 018 paclitax	Medtronic inc.	Approved 05/29/14	FP	Paclitaxel 3.5 μg/mm^2^	Urea
Lutonix drug coated balloon PTA catheter	Lutonix	Approved 10/09/14	FP	Paclitaxel 2 μg/mm^2^	Polysorbate and sorbitol

## Drugs used in endovascular device coatings and their cellular targets

### Paclitaxel

Paclitaxel, a potent tumoricidal drug, was first isolated in 1967. Paclitaxel has been used extensively as a cancer therapy. However, research performed in the 1990s demonstrated the ability of paclitaxel to inhibit VSMC proliferation [35]. The first paclitaxel-eluting stent was approved by the FDA for use in the coronary arteries in 2004 after promising results from the TAXUS trials [36]. However, concerns regarding permanent stents and their association with IH lead to the development of paclitaxel coated balloons for coronary angioplasty. In the 2010s, paclitaxel-coated balloons underwent clinical trials to evaluate their use in small coronary arteries [37]. In 2015, the first trial using a Paclitaxel-coated DCB in FP arteries followed [38]. Since then, paclitaxel has been used commonly as a DCB coating. Paclitaxel acts as a microtubule stabilizing agent that prevents the tubular migration necessary for mitotic spindle assembly and causes cell cycle arrest in the G2/M phase [39]. Ultimately, these arrested cells undergo apoptosis. Paclitaxel upregulates BCL-2, DAP3, BAX, DAD1, and several other pro-apoptotic genes [40]. Additionally, paclitaxel affects multiple pathways associated with cellular proliferation, including receptor tyrosine kinases (RTK), TGF-B, and upstream regulators of the ERK pathway [40]. The effects of paclitaxel are non-specific; while paclitaxel inhibits VSMC proliferation, it may delay reendothelialization of denuded epithelia [41].

#### Paclitaxel controversy

With respect to decreased IH and reduced restenosis rates, the use of paclitaxel-coated devices represents a significant improvement over BMS and POBA. However, the use of paclitaxel in DES and DCB has not been without controversy. Katsanos et al. conducted a meta-analysis including 28 research-controlled trials assessing the use of paclitaxel-eluting DES and DCB. Their study demonstrated an increased risk of mortality associated with paclitaxel-coated devices [42]. A follow up meta-analysis demonstrated no significant increase in all-cause mortality between 1 and 2 years, but an increased risk of mortality between year 3 and 5 [43]. In 2019, the FDA issued a letter to healthcare providers to notify them of the increased late mortality signal. Following this, manufacturers of FDA-approved devices submitted deidentified individual patient data to the VIVA Physicians medical research organization, which produced an aggregate meta-analysis published in Circulation in May 2020 stating that no increased mortality signal was found [44]. Further meta-analyses of clinical trial data found no increase in all-cause mortality [45]. The FDA also analyzed data from several trials including the VOYAGER PAD study, the BARMER Health Insurance study, the Medicare Safe-PAD study, the U.S. Veterans Health Administration study, and the SWEDEPAD interim analysis [46]. By July 2023, the FDA issued updated guidance stating there was no increased mortality signal.

### Sirolimus

Sirolimus, also known as rapamycin, is a macrolide antibiotic with poor antibacterial capabilities that is used as an immunomodulatory, cytostatic, and antiproliferative agent. Sirolimus acts to inhibit the mTOR pathway by reversibly binding to FK506-binding protein 12 (FKBP12). FKBP12 binds tacrolimus (FK506) as well as rapamycin and other rapalogs, creating a complex that inhibits MTORC1 [47]. The downstream result of mTORC1 inhibition is that cells cannot progress through the G1/S transition and are maintained in G1. Sirolimus inhibits VSMC proliferation via this mechanism. Additionally, inhibition of NF-kB by sirolimus has been previously demonstrated [48]. Inhibition of NF-kB has downstream effects on the expression of leukocyte adhesion molecules and chemoattractants, conferring an anti-inflammatory role in addition to its other effects. While sirolimus and paclitaxel are some of the most frequently used drugs in DCBs, there are key differences in how they affect cellular processes Table 2.

**TABLE 2:** Paclitaxel versus sirolimus: Reported effects exerted on cellular events that follow endovascular interventions.

Cellular events that follow intervention with stents and balloons	Paclitaxel	Sirolimus
Platelet aggregation	Inhibits collagen-mediated platelet aggregation and TXA_2_ synthase [49]; enhances platelet aggregation via increased sensitivity to ADP [50]	Enhanced platelet aggregation via increased platelet sensitivity to ADP [51, 52]
Inflammation	Increased [53, 54]	Reduced [55, 56]
VSMC proliferation	Inhibited [35]	Inhibited [57]
VSMC migration	Inhibited [35]	Inhibited [58]
Cell death	Promotes apoptosis and autophagy [39, 40, 59]	Prolongs the G1 phase prior to the G1/S checkpoint in a reversible manner [47]
Reendothelialization	Inhibited [60]	Inhibited [61]

### Everolimus and other limus drugs

Everolimus, 40-O-(2-hydroxyethyl)-rapamycin, is a sirolimus analog with a hydroxyethyl group at C-40 [62]. Modification of sirolimus in this manner was intended to improve oral bioavailability but resulted in several key differences in the behavior of everolimus [63]. Everolimus is an mTOR inhibitor with a weaker binding affinity for FKBP12 than sirolimus. Unlike sirolimus, which only inhibits MTORC2 with chronic use, everolimus demonstrates activity against both MTORC1 and MTORC2 [64]. Similar to sirolimus, the end result is that cells do not progress past G1 of the cell cycle [65].

Everolimus was initially developed for use in solid organ transplants. Owing to its antiproliferative properties, interest in its use in coronary stents culminated in the FUTURE and SPIRIT FIRST clinical trials [66, 67]. In 2009, the FDA approved the first everolimus-eluting stent for use in the coronary arteries. Since then, everolimus-eluting stents have been designed for use in peripheral arteries, including the XIENCE Prime™ BTK Everolimus Eluting Peripheral Stent by Abbott (currently marketed outside of the US). Most recently, the Esprit™ BTK everolimus eluting resorbable scaffold system by Abbott Vascular was approved by the FDA for use in infrapopliteal arteries.

Zotarolimus is the first limus drug designed specifically for use in drug-eluting stents. Some of these stents include the Medtronic Endeavor stents and now the Medtronic Onyx Frontier stent, which are used in CAD [68, 69]. Zotarolimus exhibits enhanced lipophilicity compared to sirolimus, allowing it to traverse cellular membranes more easily than the less lipophilic drugs sirolimus and paclitaxel [70]. As in sirolimus and everolimus, zotarolimus inhibits smooth muscle cell proliferation via mTOR inhibition [71]. A study assessing reendothelialization rates in an ilio-femoral atherosclerotic rabbit model treated with zotarolimus-compared to everolimus-eluting stents found decreased inflammation and increased expression of CD31, a marker of mature endothelial cells, in the everolimus-treated group compared to the zotarolimus-treated group, suggesting that reendothelialization may occur faster with everolimus than zotarolimus [72]. However, an *in vitro* study examining reendothelialization after inducing an injury in cultured endothelial cells found that regrowth of the injured area occurred more quickly with zotarolimus compared to sirolimus or paclitaxel [73]. While zotarolimus eluting stents have been used in CAD, this technology is not FDA approved for peripheral arteries. Similarly, zotarolimus-coated balloons have been evaluated in pre-clinical studies using the swine femoral artery, however there are no zotarolimus-coated balloons that are FDA approved for treating PAD lesions [74].

## Coating technologies and their contributions to efficacy and/or complications

### Contributions to efficacy

Unlike DES, which remain in the vessel indefinitely, DCB must be designed such that the coating adheres to the balloon during tracking while also effectively releasing all of their drug/carrier cargo to the target lesion within a window of 1–3 min. Therefore, the development of effective transfer mechanisms is emphasized in DCB development. Additionally, the coating should have a uniform density and elicit minimal inflammatory responses. The coating should also enhance drug transfer from the device to target tissues with effective tissue penetration and appropriate tissue residence time. Coatings often include excipients, which are defined as ingredients other than the active drug within a formulation. Commonly used excipients in DCBs include urea, polyethylene glycol (PEG), polysorbate and sorbitol, iopromide, and others [75–78]. Excipients can improve drug stability within the vessel environment and modify tissue uptake. Excipients can also act as delivery vehicles, directly transporting drugs to target sites [79–81].

Additional considerations are the hydrophilicity or hydrophobicity of the coating. Hydrophobic coatings create a repellent surface that allows blood to pass over the device. However, hydrophobic coatings may be less hemocompatible than hydrophilic coatings. Multiple studies have associated complement component C3 and fibronectin adsorption as well as monocyte and platelet adhesion with hydrophobic coatings [82–84]. On the other hand, recent investigations into superhydrophobic coatings demonstrate enhanced hemocompatibility. Experimental studies assessing the hemocompatibility of superhydrophobic coatings show reduced protein adsorption and decreased platelet adhesion [85–87].

Polymeric coatings have been used extensively on medical devices. Polymeric hydrophilic coatings exhibit less blood protein adsorption than polymeric hydrophobic coatings, demonstrating enhanced hemocompatibility [82]. Polymeric hydrophilic coatings attract water and tend to be more slippery than hydrophobic coatings, which promotes navigation through tortuous arterial segments during PTA procedures. Additionally, hydrophilic coatings have been shown to promote rapid drug transfer [88]. In DCBs where the drug load needs to be transferred rapidly from the balloon surface to the treatment site, hydrophilic coatings are advantageous. The downside of hydrophilic coatings is that while they promote drug transfer, they are also prone to significant drug loss from the coating surface during catheter tracking [89, 90].

**FIGURE 2:**
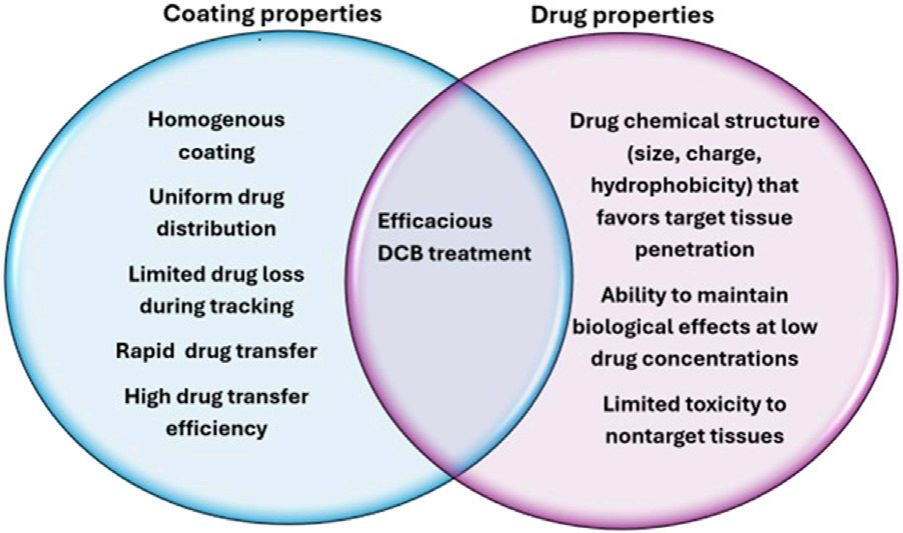
Ideal characteristics of DCB coatings and drugs to maximize efficacy.

Aside from the physical properties of the coating, the coating method itself is also an important factor that contributes to efficacy. Several methods exist for applying coatings, each with their own benefits and drawbacks. There are many methods including dip coating, air or ultrasonic spray coating, and others. A study conducted by Gandhi and Murthy found that dip coating balloon catheters created a generally smooth surface, but the coating accumulated around pleated regions of the uninflated balloons, which could alter drug release. The same study showed that an ultrasonic spray coating created a balloon surface with microcracks, while airbrushing created the most uniform surface [91]. Ideal properties of coating and drugs used for DCB are summarized below Figure 2.

### Coating distribution, dose, composition and their relations to complications

The interactions between active drugs and the coating are critical components of not only drug delivery, but also the biocompatibility of combination devices. While various excipient coatings have markedly improved drug delivery, coating embolization is also a significant concern in endovascular devices. A study performed by Torii and colleagues compared DCB coating embolization in swine arteries using the IN. PACT, Ranger, and Stellarex DCBs. The IN. PACT DCB utilizes a highly hydrophilic urea coating. In contrast, the Ranger DCB uses an acetyl tributyl citrate carrier that is highly hydrophobic. The study concluded that downstream emboli were found more frequently in the IN. PACT DCB treated arteries [92]. While correlations were drawn between increasing paclitaxel concentration and increased incidence of emboli, it is also possible that the hydrophilicity of the IN. PACT DCB urea coating contributed to embolization via loss during tracking or failure to adhere to the vessel wall due to enhanced affinity for the hydrophilic blood compartment compared to hydrophobic cellular membranes [93]. Previous work in porcine models demonstrated distal excipient-crystalline drug emboli present in the coronary band of pigs after femoral artery angioplasty with paclitaxel-coated balloons [94].

Biodegradable polymers were designed to mitigate inflammatory and thrombotic responses associated with early durable stent coatings. However, biodegradable polymers have also been associated with biocompatibility issues. In the multi-center study conducted by van der Giessen and coworkers, coating materials consisting of both durable and biodegradable polymers were applied to stents that were implanted into pig arteries and left in place for 4 weeks. All of the polymers tested, including biodegradable PLGA, elicited inflammation in the implanted arteries and lumen narrowing was observed [95]. However, the stents themselves could have contributed to the elicited reaction, as the materials were not sterilized prior to implantation. They also observed that the applied polymer coatings did not fully cover the stents after expansion, leaving areas of bare metal in direct apposition to the treated arteries [95]. Other cited drawbacks associated with PLGA are bulk polymer erosion and burst drug release [96]. Additionally, accumulation of glycolic and lactic acid at local sites due to rapid breakdown of PLGA may incite inflammatory responses [97]. Meanwhile, other studies have found PLGA coated stents to be no more inflammatory than BMS [98]. While PLGA is one of the most widely used polymers in medical devices, continued investigations into PLGA biocompatibility are warranted.

Submicron drug coatings including polymeric nanoparticles are being developed for treatment of PAD lesions [32, 99]. While these technologies have been studied in the setting of cancer treatment and have shown promising results, there are points to consider with the use of submicron particles. Adherence of nanoparticles to the balloon surface during delivery is key; otherwise, there is potential for blood flow to cause early deployment of particles to non-target areas. Blood interactions with nanoparticles are important considerations. Nanoparticle composition, size, and charge are determining factors for how the particles interact with cells in the blood compartment. For example, carbon nanoparticles have been shown to promote venous thrombosis and platelet aggregation [100]. Additionally, some nanoparticles can enter cells such as RBCs by direct penetration of the cell membrane [101]. While carbon nanoparticles have been shown to promote thrombosis, other studies have documented a lack of increased platelet aggregation with the use of PLGA nanoparticles, highlighting the importance of particle type selection for use in blood contacting devices [102]. Bakhaidar and coworkers demonstrated that PLGA-PEG nanoparticles ranging from 112 to 576 nm interacted with and bound to platelets; however, this did not increase platelet aggregation [103].

## Emerging devices in BTK PAD therapy

### Emerging drug coated balloons

While there have been several clinical trials investigating DCBs for use in BTK PAD lesions, none have emerged as FDA-approved. DCB treatment has not yet demonstrated long term benefits over POBA treatment alone in clinical trials. However, several new DCB have been granted breakthrough device designation and are currently in clinical trials. These newer DCB formulations range from microcrystalline polymer-free coatings to liquid delivery systems. These combination devices deliver sirolimus as well as combinations of sirolimus and paclitaxel. A table summarizing these novel devices, what we know about their coating properties, and selected clinical trials is listed below Table 3.

**TABLE 3:** Recent DCBs with FDA breakthrough designation for BTK PAD and associated clinical trials.

Device	Drug	Coating	Clinical trials
Sundance™ DCB, surmodics inc	Microcrystalline sirolimus *Coating density undisclosed*	*Undisclosed*	SWING
Selution™ SLR, cordis	Sirolimus 1 μg/mm^2^	PLGA (poly (lactic-co-glycolic acid)) microspheres	PRESTIGE PRISTINE SUCCESS SELUTION4BTK
MagicTouch™ DCB, concept medical	Sirolimus 1.27 μg/mm^2^	Sub-micron phospholipid carrier	XTOSI FUTURE-BTK LIMES MAGICAL BTK SIRONA
SirPlux duo Advanced NanoTherapies	Co-encapsuled 1:9 paclitaxel:sirolimus w/w *Coating density undisclosed*	Nanoparticle, carrier composition undisclosed	ADVANCE-DCB

The MagicTouch DCB is used in coronary applications in Europe and Asia; however, it has yet to gain FDA approval in the United States. Recently, Concept Medical’s MagicTouch DCB received FDA breakthrough designation for BTK PAD lesions. This DCB utilizes a polymer-free approach with Nanolute technology, proprietary 100–300 nm phospholipid microspheres carrying sirolimus. The coating density of the MagicTouch DCB is 1.27 μg/mm^2^ [104]. Three-year results from the XTOSI pilot study published in 2024 demonstrate 77.8% freedom from major amputation for BTK lesions [105]. Several clinical trials are currently investigating the use of MagicTouch DCB in both FP and BTK PAD.

Like the MagicTouch DCB, the Sundance™ DCB by SurModics utilizes a polymer-free formulation. The Sundance formulation is a microcrystalline sirolimus coating with their coating density and proprietary excipient yet to be disclosed. In 2020, they commenced the SWING study, a prospective multi-center single arm study that enrolled 35 patients. The completion date for the study was 30 January 2024. SurModics has yet to publish the results of their study, although they reported 71.4% primary patency maintained at 24 months [106].

The Selution SLR DCB uses sirolimus-loaded PLGA microspheres contained in MicroReservoirs which are coated with Cell Adherent Technology (CAT), a mixture of phospholipids that reportedly protect the microspheres during catheter insertion and tracking. The coating density of the balloon is 1 μg/mm^2^ [107]. The PRESTIGE pilot study investigated the performance of the Selution SLR DCB in occlusive tibial disease and showed 81.5% tibial patency at 6 months [108]. In 2023, the prospective, randomized multicenter single blinded study, SELUTION4BTK completed enrollment with 377 subjects. The aim of the study is to assess the safety and effectiveness of the Selution SLR DCB in treating BTK PAD in patients with CTLI. The anticipated completion date is 30 July 2029 [109]. In May 2025, investigators reported 12-month data from SUCCESS PTA study at the 2025 New Cardiovascular Horizons meeting. SUCCESS PTA is a single arm post-market surveillance study conducted out of treatment centers in Europe. They reported 2.2% target limb amputation and greater than 90% freedom from clinically driven target lesion revascularization in the 12 months cohort, with an average lesion length of 12–13 cm [110].

While the use of paclitaxel in DCB products is generally being replaced by sirolimus in new generation products, the SirPlux Duo by Advanced NanoTherapies combines both sirolimus and paclitaxel in a novel dual-agent formulation. Paclitaxel and sirolimus are co-encapsulated at a 1:9 w/w ratio within nanoparticles [32]. The coating density, excipient, and composition of the nanoparticle carrier are unclear, although a patent submitted by Advanced Nanotherapies in 2023 suggests that PLGA may be used to entrap paclitaxel and sirolimus [111]. Preclinical work in porcine coronary and femoral arteries and rabbit iliac arteries demonstrate reduced VSMC proliferation with SirPlux Duo compared to paclitaxel DCB treatment [32]. They also investigated particle embolism in a porcine coronary artery model and found a significant reduction in embolized material with the SirPlux Duo DCB. A first-in-human clinical trial investigating the use of SirPlux Duo in patients with *de novo* CAD lesions is currently ongoing [112]. Listed below are some of the early results and up-and-coming clinical trials for DCBs with breakthrough designation status for BTK disease Table 4.

**TABLE 4:** BTK trial data for DCB with FDA breakthrough designation status.

Device	Clinical trial	Trial type	Primary patency	CD-TLR (or freedom from CD-TLR)	Freedom from major amputation
Sundance™ DCB, surmodics inc.	SWING [113] (12 months results)	Single arm feasibility study	80%	8%	Not reported
	SWING [106] (24 months results)	Single arm feasibility study	71.4%	8.3%	Not reported
Selution™ SLR, cordis	PRESTIGE [108] (12 months results)	Single arm pilot study	78%	93% freedom from CD-TLR	87% at 12 months
	PRISTINE [114] (12 months results)	Single arm registry study	59.5%	7.4%	72.6% (amputation free survival)
MagicTouch™ DCB, concept medical	XTOSI [105] (36 months results)	Single arm pilot study	50% (at 24 months)	77.8% freedom from CD-TLR	81% at 36 months
Pending trials, results not yet reported:					
Selution™ SLR, cordis	SELUTION4BTK [115]	Randomized controlled trial vs. POBA			
MagicTouch™ DCB, concept medical	FUTURE-BTK [116]	Randomized controlled trial vs. POBA			
	LIMES [117]	Randomized controlled trial vs. POBA			
	MAGICAL BTK [118]	Randomized controlled trial vs. POBA			

### Other balloon-based therapies and novel lesion preparation devices

Aqueous delivery systems that circumvent concerns surrounding the use of drug-coated surfaces in the blood compartment have also been investigated. Atigh et al., delivered paclitaxel via liquid delivery in saline with iohexol as the excipient in *ex vivo* porcine carotid arteries using the Occlusion Perfusion Catheter system by Advanced Catheter Therapies [119]. They were able to demonstrate that the Occlusion Perfusion Catheter effectively delivered the liquid drug into the arterial wall. Similarly, the Virtue SAB by Orchestra BioMed uses a novel AngioInfusion balloon that delivers lyophilized submicron sirolimus in a polyester nanoparticle carrier via aqueous delivery. This device received breakthrough designation status for BTK PAD, and most recently, investigational device exemption by the FDA in May 2025. Currently, Orchestra Biomed plans to start the VIRTUE trial, a pivotal clinical trial looking at coronary in-stent restenosis.

While not strictly a balloon device, the novel Spur Retrievable Stent System by Reflow Medical has potential to enhance DCB therapy. This device consists of a self-expanding, balloon-delivered stent covered in radial spikes which penetrate the arterial wall. By creating channels in the arterial wall, the Spur device disrupts calcification and enhances vessel compliance, reducing elastic recoil immediately after treatment [120]. Additionally, these arterial channels may increase DCB drug penetration. Previously completed clinical trials investigating the use of the Spur BTK followed by DCB therapy include the DEEPER [121], DEEPER OUS [120], and most recently, the DEEPER LIMUS [122] trials.

### Stents and resorbable scaffolds

While this review focuses primarily on balloon-based therapies, we would be remiss to exclude these devices from discussion. While the “leave nothing behind” approach has favored DCB for BTK disease, stents and resorbable scaffolds have been gaining traction. Several stents and resorbable scaffolds have gained FDA breakthrough designation status, and one resorbable scaffold, the Esprit BTK everolimus-eluting bioresorbable scaffold by Abbott, recently gained FDA approval [123].

Resorbable scaffolds aim to bridge the gap between providing structural support and minimizing permanent implants. In April 2024, Abbott’s Esprit BTK everolimus-eluting bioresorbable scaffold received FDA approval [123] for CTLI in BTK disease. According to Abbott, this bioresorbable stent maintains radial strength similar to metal stents for the first 6 months and fully dissolves within two to 3 years. In the LIFE-BTK trial, Abbott reported substantially improved efficacy compared to POBA with respect to primary efficacy endpoints at 1 year [124]. Two-year follow up demonstrated continued superior efficacy over POBA, with respect to a composite of limb salvage and primary patency [125].

In March 2024, the Biotronik Freesolve BTK RMS received breakthrough designation status for CTLI BTK disease. This resorbable metal scaffold consists of a proprietary magnesium alloy utilizing sirolimus to treat lesioned arteries [126]. The first-in-human BIOMAG I trial examined late lumen loss (LLL) as a primary endpoint in coronary artery disease. Between 6 and 12 months, significant increase in in-device LLL was reported, however no scaffold thrombosis was observed [126]. The BIOMAG II RCT trial began enrollment in in May 2024, and will compare safety and efficacy endpoints versus the Xience everolimus-eluting stent [127].

Efemoral medical’s Efemoral Vascular Scaffold System also received breakthrough designation status in 2024. This bioresorbable system, like the Freesolve, utilizes sirolimus as an antirestenotic agent. The Efemoral vascular scaffold system focuses on enhancing biomechanical compatibility, utilizing a patented FlexStep system which utilizes interscaffold spaces to enhance device flexibility [128]. The first-in-human EFEMORAL I trial is still ongoing (NCT: 04584632).

Another notable stent to receive FDA breakthrough designation status for BTK indications includes Elixir Medical’s DynamX BTK system. The DynamX BTK Bioadaptor represents a hybrid between bioresorbable polymer elements and metallic scaffolding materials which deploy as the polymer dissolves [129]. The bioresorbable coating consists of poly-L-lactide (PLLA) eluting novolimus [129] and is currently undergoing clinical trials for coronary applications, with plans to design a modified version of the device for BTK therapy [130].

## Discussion

PAD is a complex and multifactorial disease that leaves patients prone to thrombosis, arterial occlusion, and loss of limb. Endothelial injury and dysfunction play a key role in the pathogenesis of PAD. PAD below the knee is challenging to treat due to the types of lesions, extent of lesions, as well as significant problems with calcification as a barrier to drug penetration and vascular elastic recoil limiting luminal diameter post-treatment. Yet, numerous novel devices are on the horizon. While DCBs for use below the knee have yet to gain FDA approval, lessons learned from previous device iterations pave the way forward for next-generation devices. Further advancement in coating technology and drug delivery systems permit the use of less drug than older generation devices and rely less on large particulate and crystalline coatings. These changes may limit toxicity off target associated with commonly used drugs like paclitaxel and sirolimus. Drug eluting stents and bioresorbable scaffolds have been gaining momentum in BTK disease treatment and provide another promising avenue for interventions. Abbott’s Esprit BTK received FDA approval, and several bioresorbable scaffolds and DES received FDA breakthrough designation status in just the past year. As clinical trials progress, we will discover whether these breakthrough therapies can gain FDA approval for BTK disease treatment.
